# Tumor necrosis factor (TNF)-α- 308 G/A gene polymorphism (rs1800629) in Egyptian patients with alopecia areata and vitiligo, a laboratory and *in silico* analysis

**DOI:** 10.1371/journal.pone.0240221

**Published:** 2020-12-28

**Authors:** Talal Abd El-Raheem, Rania H. Mahmoud, Enas M. Hefzy, Mohamed Masoud, Reham Ismail, Nesreen M. M. Aboraia

**Affiliations:** 1 Department of Dermatology, STDs, and Andrology, Faculty of Medicine, Fayoum University, Fayoum, Egypt; 2 Department of Medical Biochemistry and Molecular Biology, Faculty of Medicine, Fayoum University, Fayoum, Egypt; 3 Department of Medical Microbiology and Immunology, Faculty of Medicine, Fayoum University, Fayoum, Egypt; 4 Department of Public Health, Faculty of Medicine, Fayoum University, Fayoum, Egypt; 5 Manshiat Albakry General Hospital, Cairo, Egypt; San Gallicano Dermatologic Institute, ITALY

## Abstract

**Purpose & methods:**

Several single-nucleotide polymorphisms (SNPs) in the promoter region of the TNF-α gene can cause variations in the gene regulatory sites and act as risk factors for some autoimmune disorders as alopecia areata (AA) and vitiligo. This study aimed to detect the serum TNF-α (sTNF) level (by ELISA) and the rs1800629 (by real-time PCR) among AA and vitiligo Egyptian patients and to determine their relation with disease duration and severity. *In silico* analysis of this SNP to study the molecular regulation of the mutant genotypes was also done.

**Results:**

In AA patients, no risk was associated with the mutant genotypes *vs*. the normal genotype, or with A allele *vs*. G allele. The risk of vitiligo was significantly higher with the G/A and A/A genotypes compared with HCs (*p* = 0.011). Similarly, a significantly increased risk was noted in patients with A allele *vs*. G allele *(p*<0.0001). In AA and vitiligo patients, a significant increase in sTNF-α levels was noted in the mutant G/A genotypes *vs*. the normal G/G genotype (*p*<0.0001) and in the A allele vs the G allele *(p*<0.0001). According to the *in silico* analysis, this SNP could mainly affect the SP1 transcription factor binding site with subsequent effect on TNF-α expression.

**Conclusion:**

According to results of the laboratory and the *in silico* study, the mutant TNF-α (308) genotypes were risk factors that conferred susceptibility to vitiligo among Egyptian patients but had no effect on the susceptibility to AA.

## 1. Introduction

Alopecia areata (AA) is an inflammatory disease that affects hair follicles. Clinically, the disease manifests as well-circumscribed patches of hair loss [1]. Vitiligo is an acquired disorder characterized by the improper function of the epidermal melanocytes [2]. Vitiligo and AA are remarkably similar in pathogenesis that has been attributed to both innate and adaptive immune system disorders. In both diseases, augmented reactive oxygen species and expanded cellular stress have been reported as the triggering factors of the innate immune system [3]. Additionally, risk alleles that affect both innate and adaptive immunity in both diseases have been detected by genome-wide association studies [4, 5]. Most notably, studies in mouse models of vitiligo and AA have specifically linked an abnormal IFN-γ-driven immune response as the main inducers of the pathogenesis of these diseases [4, 6].

Tumor necrosis factor-α (TNF-α) is a significant multi-functional cytokine produced by macrophages, fibroblasts, keratinocytes, and T-lymphocytes with a wide range of biological activities [7]. TNF-α is recognized to play a major role in the pathogenesis of AA. Previous studies have reported that TNF-α, in conjunction with IL-1α and IL-1ß, decreases the size and causes vacuolation of the matrix cells of follicular bulb, causes disorganization of the follicle melanocytes together with abnormal keratinization and differentiation of the inner root sheath and the precortical cells [8]. TNF-α has a key role in the pathogenesis of vitiligo as it affects the apoptosis of melanocytes. Also, in the epidermis, the keratinocytes make cytokines, as TNF-α, IL-1α, IL-6, and TGF-β, which inhibits melanocyte proliferation and melanogenesis. Besides, TNF-α can prevent melanocyte stem cells development and differentiation. TNF-α has a suppressive action on tyrosinase and tyrosinase-related proteins that inhibit melanogenesis [7].

Several single-nucleotide polymorphisms (SNPs) have been recognized in the promoter region of the human TNF-α gene. These SNPs can cause structural variations in the regulatory sites of the gene which may affect the production or function of TNF-α [7]. The TNF-α-308 guanine/adenine (G/A) (rs1800629) was reported to have a major effect on TNF transcription in macrophages [9]. There are two alleles at this polymorphic site, TNF-α-308G, and TNF-α-308A. In normal populations; TNF-α-308G homozygosity is the predominant genotype [10]. The TNF-α-308 polymorphism is one of the most important TNF polymorphisms in human disease susceptibility such as; autoimmune hepatitis, type 1 and type 2 diabetes mellitus, acne vulgaris, psoriasis, and Sjogren's syndrome [11].

Previous studies have examined the potential contributions of the TNF-α-308 G/A polymorphism to the susceptibility to AA [12] and vitiligo [13]. However, the findings of these studies have been inconsistent, likely because of the small sample sizes and the low statistical power. Besides, allelic frequencies in genes usually vary significantly between geographical regions and ethnic groups accordingly specific association studies are needed to determine genetic associations in different countries and ethnicities [13], which necessitates replication studies to be performed between different ethnic groups.

The current study aimed to measure the serum TNF-α (sTNF-α) level and the role of the SNP (rs1800629) as a risk for the susceptibility to AA and vitiligo among Egyptians, and to determine its correlation with duration and severity of these diseases. Another objective of this study was to use bioinformatics to analyze this SNP and to demonstrate its effect and interaction with factors that regulate the expression of this gene.

## 2. Materials and methods

### 2.1. Study population

This case-control study has included 225 subjects, 75 AA patients, 75 vitiligo patients, and 75 age and sex-matched healthy controls (HCs). Patients were recruited from the outpatient clinic, Department of Dermatology, faculty of medicine, Fayoum University. Patients with any other evident skin disorders, systemic or autoimmune diseases, or had hair loss or leukoderma secondary to other causes, and those under immunosuppressive drugs treatment or PUVA (psoralen and ultraviolet A) for 3 months before the beginning of the study were excluded.

The sample size was calculated using G*Power version 3.1.9.4. A minimal sample size of 64 subjects in each group was needed for a power level of 0.80, alpha level of 0.05 (two-tailed), and a medium effect size of 0.50 for the sTNF-α level. To overcome the problem of missing values, the calculated sample size was increased by 15% to reach 75 participants in each group.

### 2.2. Clinical assessment of subjects

(A). All patients were subjected to complete personal, disease, medical and family history taking. They also had a full dermatological examination to find out the type, extent, and sites of the lesions according to the Severity of Alopecia Tool (SALT) score for AA patients [14] and Vitiligo Area Scoring Index (VASI) score for vitiligo patients [15].

#### Clinical assessment of AA patients

According to the combination between the density and extent of scalp hair loss, the SALT score in AA patients was calculated [16]. Patients were classified according to the severity of AA into: Mild AA (S1) with SALT score (<25% hair loss), Moderate AA (S2) with SALT score (25–49% hair loss), and Severe AA (S3, S4, a, b and S5) with SALT score (>50% hair loss) [17].

#### Clinical assessment of vitiligo patients

The total body VASI was concluded using a method that includes proportions from all regions of the body with a possible range, 0–100 [15]. One hand unit, which encompasses the palm plus the volar surface of all the digits, is approximately 1% of the total body surface area and is used as a guide to estimate the baseline percentage of vitiligo involvement in each body region [18]. The extent of residual depigmentation is expressed as previously described by **Kawakami and Hashimoto, (2011)** [15].

(B) All HCs had a complete history taking and medical examination to exclude any skin or systemic diseases.

### 2.3. Collection and storage of blood sample

Venous blood samples were collected from all subjects and were divided into two parts. The first part was put in a centrifuge tube and was incubated for 15 minutes at 37°C then centrifuged at 3.000 rpm for 10 minutes. Then sera were separated and stored at -80°C till used in the estimation of sTNF-α level by ELISA technique. The second part was put in an EDTA tube for DNA extraction from the whole blood and was stored at -80°C until the analysis of the rs1800629 by real time-PCR (RT-PCR).

### 2.4. Assay of the serum TNF-α level

All study groups were subjected to assessment of sTNF-α level on the same day to reduce inter-assay variations. This was done by human TNF-α ELISA Kit provided by Koma biotech, INC, Seoul, Korea., according to the manufacturer's protocol.

### 2.5. Analysis of TNF-α -308 (G/A) gene polymorphism

DNA was extracted from whole blood using the QIAamp Extraction Kit (QIAamp; Qiagen, Germany) according to the manufacturer’s instructions. All samples of extracted DNA were stored at −80°C until analysis. Genotyping was achieved by RT-PCR using the TaqMan allelic discrimination assay; [TaqMan TNF-α SNP genotyping assays, numbers C_7514879_10 (rs1800629 G/A) purchased from Applied Biosystems, Foster City, CA, USA] according to the manufacturers' protocol. Briefly, assays were performed in a total reaction volume of 25μl containing 12.5μl of 2X TaqMan^®^ Universal Master Mix, 1.25 μl of 20X working mix of SNP genotyping assay and 1μl of genomic DNA. The RT-PCR was performed on the Rotor gene Q Real Time PCR System (Qiagen, Valencia, CA, USA). The thermal cycling profile was 10 min at 95°C followed by 45 cycles at 92°C for 15 s then 60°C for 60 s for denaturation, annealing and extension steps. After completion of the reaction, the amplification curve was verified and all genotypes were detected by reading the results in SDS v2.1 and RQ Manager 1.2 software.

### 2.6. *In silico* effect of rs1800629 SNP on TNF-α gene

*In silico* genetic analysis of SNPs is an easy and affordable method that can facilitate genetic studies. Few *in silico* studies have addressed TNF-α gene mutations, and fewer have focused on SNPs. SNPs may rigorously affect the function of the transcription factors (TFs). All data related to rs1800629 SNP was gathered from dbSNP in National Center of Biotechnology Information (NCBI) database (https://www.ncbi.nlm.nih.gov/snp/). It was analyzed by using Regulome DB, HaploReg and Alibaba softwares.

#### Alibaba 2.1

Alibaba algorithm anticipates the binding sites of the putative transcription factors which were found within the empirically defined proximal promoters. It is available at: (http://gene-regulation.com/pub/programs/alibaba2/index.html), and stem from the TRANSFAC 3.5 database [19].

#### HaploReg v4.1

HaploReg website is used to interpret the non-coding genome at variants on haplotype blocks, as SNPs at disease-associated loci. Using the linkage disequilibrium (LD) data from the 1000 Genomes Project, small indels and linked SNPs can be seen together with protein binding sites and chromatin state from the ENCODE and Roadmap Epigenomics projects. HaploReg is available at: https://pubs.broadinstitute.org/mammals/haploreg/haploreg.php [20].

#### Regulome DB

Regulome DB (available at: https://regulome.stanford.edu/regulome-search/) is a database that mark up SNPs with predicted and well-known and regulatory factors in the intergenic regions of the Homosapiens genome. The predicted and well-known regulatory DNA factors include regions for attachment sites of the transcription factors, DNase hypersensitivity, and the promoter regions that have been characterized to regulate transcription. Public datasets from the ENCODE project, GEO, and published literature were the sources of these data [21].

### 2.7. Ethical approval

The study was approved by the research ethical committee, Faculty of Medicine, Fayoum University (Research M171, committee session n.26, June, 2016). Informed written consent was collected from all the enrolled subjects after a detailed, simplified explanation of the study.

### 2.8. Statistical analysis

Deviation from Hardy-Weinberg equilibrium (HWE) was tested for each polymorphism using a Chi-square test by a specific calculator; available online at http://www.oege.org/software/hardy-weinberg.html. The collected data were statistically examined using SPSS (software statistical computer package) version 22 (SPSS Inc, USA). For quantitative data, the mean, median, standard deviation (SD), and interquartile range (IQR) were calculated. Kolmogorov-Smirnov test (KS) test was performed as a test of normality. If a variable was not normally distributed, the Mann-Whitney-U test or Kruskal Wallis test was used in the comparison between any two or three groups, respectively. Otherwise, one way ANOVA was used. Qualitative data were presented as numbers and percentages- Chi-square (χ2) was used as a test of significance. Significance was adopted at *p* ≤ 0.05. Crude Odds ratio (OR) and Adjusted Odds ratio (AOR) with 95% confidence intervals (CI) for the association of the study groups with different models and allelic frequency of TNF-α-308 genotypes were calculated. Adjusted *p*-values for multiple post-hoc comparisons for the 3 groups were calculated by using the Bonferroni correction method to account for the problem of multiple testing. *P-value* (of 0.05) was divided by the number of comparisons i.e. 3 (0.05/3). Thus, test results were considered statistically significant with *p*-values ≤ 0.017. The dot graph for sTNF-α level was drawn using Graphpad Prism software 6, 2012.

## 3. Results

### 3.1. Demographic and clinical characterization of the study groups

This case-control study included 225 subjects, 75 AA patients, 75 vitiligo patients, and 75 HCs. The mean age of AA patients (mean±SD) was 24.8±18.6 years and the mean disease duration was 41.33±19.6 months. The AA patients were 30 males and 45 females. According to the SALT score, AA was mild in 56%, moderate in 33.3% and severe in 10.7% of the patients **(Table 1).** The mean age of vitiligo patients was 28.3±12.3 year and the mean disease duration was 70.5±31.8 months. VASI score ranged from 0.05–20 and mean±SD was 2.6±4.4. The clinical types, site of lesions, and course of the diseases of both patients groups is displayed in **Table 1.**

**Table 1:** Basic demographic and clinical characteristics of participants.

	AA (N = 75)		Vitiligo (N = 75)	Control (N = 75)	*p-*value
**Age (years)** Mean±SD Median(IQR)	24.8±18.6 14 (5–56)		28.3±12.3 34(6–52)	26.3±4.328(14–34)	0.175
**Males [N(%)]**	30(40.0%)		25(20.0%)	39(52.0%)	0.186
**Positive Family History**	3(4.0%)		15 (20%)		0.02*
**Disease duration Mean±SD (Months)**	41.3±19.6		70.5±31.8		<0.000*
**Course** [N(%)]					
Stable Progressive	12(16.0%) 63(84.0%)		0 75(100.0%)		<0.000*
**Clinical Type** [N(%)]					
	Single patch	30 (40.0%)	Segmented	18(24.0%)	
	Multiple patches	45(60.0%)	Non Segmented	42(56.0%)	
			Mixed	15(20.0%0	
**Site of lesions** [N(%)]					
	Frontal	42(56.0%)	Face/ neck	36(48.0%)	
	Lateral	30(40.0%)	Upper Limb	54(72.0%)	
	Back	30(40.0%)	Lower Limb	54(72.0%)	
	Body	12(16.0%)	Trunk	27(36.0%)	
**SALT Score**	**N**	**%**			
**S**					
S0	2	2.7%			
S1	40	53.3%			
S2	24	32.0%			
S3	2	2.7%			
S4	7	9.3%			
**B**					
0	66	88.0%			
1	9	12.0%			
**Grade (AA)**					
Mild	42	56%			
Moderate	25	33.3%			
Severe	8	10.7%			
**VASI score** (Mean±SD)			2.6±4.4		

* Statistical significance was considered at *p* value ≤0.05; **AA**, Alopecia areata; **IQR,** interquartile range; **SALT,** Severity of Alopecia Tool**; VASI,** Vitiligo Area Scoring Index.

### 3.2. Assay of the serum TNF-α level and its variation with the demographic and clinical characteristics of AA and vitiligo patients

#### 3.2.1. Serum level of TNF-α in the study population

**Fig 1:**
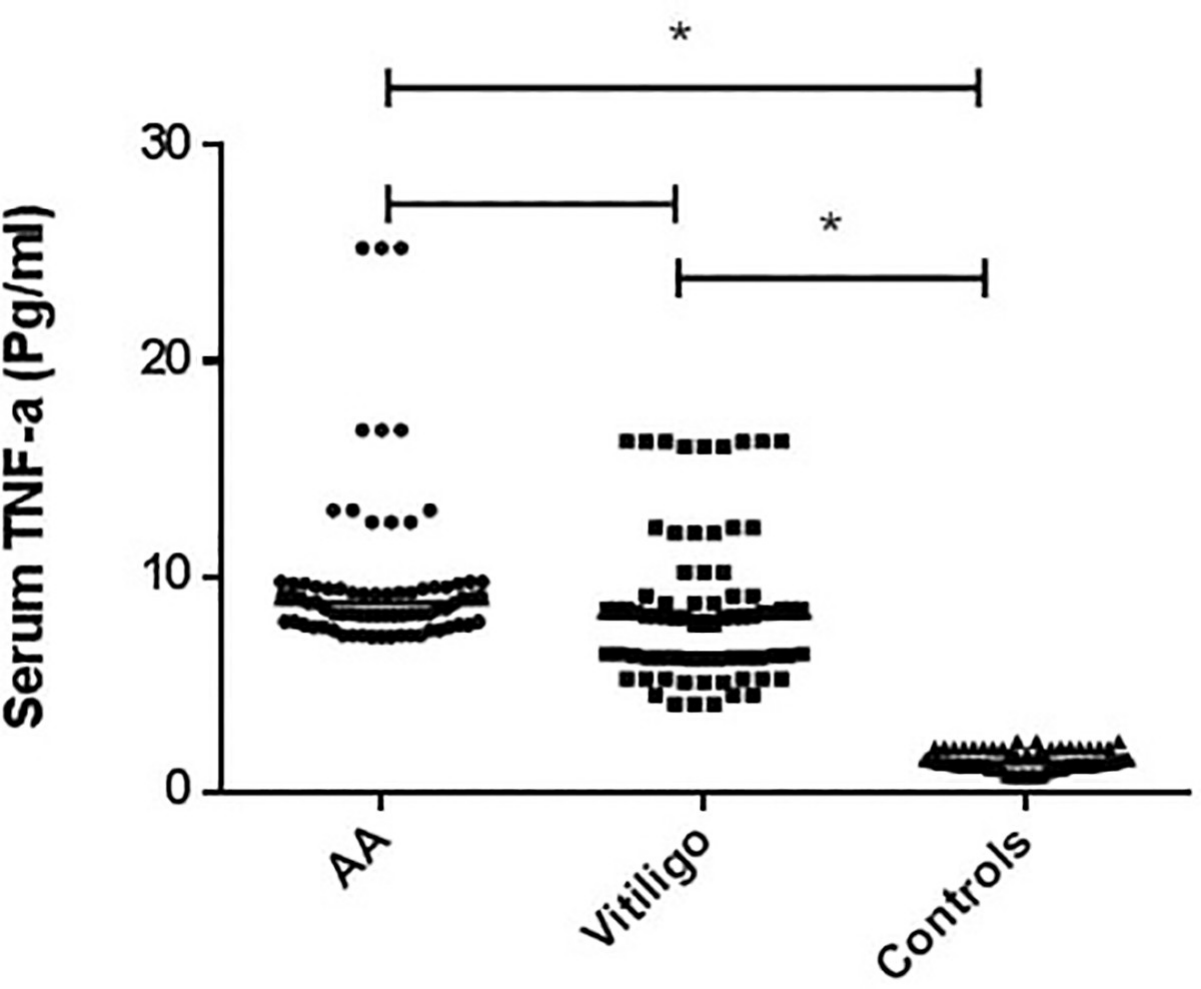
A graphpad represents a comparison of the serum TNF-α levels (pg/ml), as determined by ELISA, among vitiligo patients (n = 75), alopecia areata (AA) patients (n = 75) and healthy controls (n = 75). * Statistically significant (*p* <0.0001), Statistical significance was considered at *p* value ≤0.05. The sTNF-α level, expressed as median (IQR), was 8.8 (7.9–9.5pg/ml), 8.1 (6.3–9.1pg/ml), 1.4 (1.2–1.8pg/ml) in AA, vitiligo and HC groups, respectively. A significantly higher level of sTNF-α in AA and vitiligo patients vs the HCs was noted.

A significantly higher level of sTNF-α in AA and vitiligo patients vs the HCs was noted (*p* <0.0001). The sTNF-α level, expressed as median (IQR), was 8.8 (7.9–9.5pg/ml), 8.1 (6.3–9.1pg/ml), 1.4 (1.2–1.8pg/ml) in AA, vitiligo and HC groups, respectively **(Fig 1)**.

#### 3.2.2. Differences in TNF-α serum level concerning clinical data in AA patients

In AA patients, there was no significant difference in the sTNF-α level according to age, course of the disease, clinical presentation or the disease severity. However, the sTNF-α level was significantly higher in females than in males (*p*<0.0001). Body and back involvement and frontal lesions of the scalp were significantly associated with increased sTNF-α level (*p* = 0.013, 0.007&0.014 respectively). It also was significantly higher with SALT score B1 (*p* = 0.028) **(Table 2)**.

**Table 2:** Differences in TNF-α serum level (pg/ml) in relation to clinical data in alopecia areata patients.

		TNF-α serum level (pg/ml)		*p-*value
		Median	Range	
**Sex**	Male	7.8	7.3–8.2	<0.0001*
	Female	9.2	8.5–12.5	
**Family history**	Positive	7.3	7.2–8.3	0.004*
	Negative	8.9	7.2–25.3	
**Course**	Stable	8.7	8–11.1	0.795
	Progressive	8.8	7.9–9.5	
**Clinical**	single	8.9	8.3–9.7	0.599
	multiple	8.5	7.8–9.5	
**Site of lesions**				
**Frontal**	No	8.3	7.7–9.3	0.014*
	Yes	9.2	8.2–12.5	
**lateral**	No	9	7.7–9.7	0.733
	Yes	8.4	8.2–9.5	
**back**	No	9.1	8.3–9.7	0.007*
	Yes	8.1	7.7–9.5	
**body**	No	8.5	7.8–9.5	0.013*
	Yes	9.5	8.7–13.3	
**SALT Score**				
**S**	0	8.7	8.3–9.3	0.327
	1	8.8	7.2–13.1	
	2	8.2	7.3–25.3	
	3	10.0	9.5–10.5	
	4	9.5	8.2–16.8	
**B**	0	8.6	7.2–25.3	0.028*
	1	9.8	8.2–16.8	
**Grade of severity**	Mild	8.9	7.2–13.1	0.311
	Moderate	8.3	7.3–25.3	
	Severe	9.5	8.2–16.8	

* Statistical significance was considered at *p* value ≤0.05; **SALT**, Severity of Alopecia Tool.

#### 3.2.3. Differences in TNF-α serum level concerning clinical data in vitiligo patients

Among vitiligo patients, differences in sex, family history, clinical types of vitiligo, and site of the lesions were not associated with a statistically significant difference in the sTNF-α levels (**Table 3).** The correlation between sTNF-α and the duration of the disease and the VASI score in vitiligo patients was negative, however, this was of no statistical significance (*r* = -0.179, *p* = 0.125 *& r* = -0.014, *p* = 0.905 respectively). On the contrary, the sTNF-α levels correlation with age was positive and significant *(r* = 0.363, *p* = 0.001).

**Table 3:** Differences in TNF-α serum level (pg/ml) in relation to clinical data in vitiligo patients.

		Median	Range	*p*-value
**Sex**	Male	8.1	6.2–8.3	0.136
	Female	8.1	6.3–11.1	
**Family history**	Positive	6.4	5.3–16.3	0.952
	Negative	8.15	4.10–16.25	
**Clinical type**	Seg	8.2	4.5–8.5	0.387
	Non seg	7.3	6.3–8.8	
	Mixed	9.1	7.8–10.2	
**Site of lesion**				
**Face/Neck**	No	8.1	6.3–8.8	0.886
	Yes	8.1	5.8–9.7	
**Upper Limb**	No	8.2	4.5–8.5	0.559
	Yes	7.9	6.3–10.2	
**Lower Limb**	No	8.3	4.5–9.1	0.710
	Yes	7.9	6.3–12	
**Trunk**	No	8.1	5.7–10.4	0.551
	Yes	8.1	6.4–9.1	

### 3.3. TNF-α-308 (G/A) polymorphism (rs1800629) and its association with demographic and clinical characteristics of AA and vitiligo patients

The strength of the association between the TNF-α-308G/A polymorphism and risk of AA and vitiligo was assessed by AORs with the corresponding 95%CI for the following genetic models: 1) the genotypic model (G/G, G/A, and A/A) 2) the dominant genetic model (A/A+G/A *vs*. G/G) (A is the minor allele and G is the major allele); 3) the recessive genetic model (A/A *vs*. G/G+G/A) and 4) the A allele *vs*. the G allele analysis (data is shown in **Table 4).**

**Table 4:** Analysis of the association between TNF-α-308 G/A polymorphism and susceptibility to alopecia areata and vitiligo.

rs1800629 SNP	AA (N = 75)	Vitiligo (N = 75)	Control (N = 75)	AA vs. control	Vitiligo vs. Control
				^a^OR (95% CI), *p-*value	^a^OR (95% CI), *p-*value
	N (%)	N (%)	N (%)	^b^AOR (95% CI), *p-*value	^b^AOR (95% CI), *p-*value
**Genotypic model**				r	r
G/G	60 (80.0%)	33 (44.0%)	66 (88.0%)		
G/A	12 (16.0%)	30 (40.0%)	8 (10.7%)	^**a**^1.650 (0.631–4.311), 0.306	^**a**^7.500 (3.096–18.168), <0.0001*
				^**b**^1.761 (0.652–4.759), 0.264	^**b**^14.274 (4.661–43.708), <0.0001*
A/A	3 (4.0%)	12 (16.0%)	1 (1.3%)	^**a**^3.300 (0.334–32.588), 0.307	^**a**^24.000 (2.991–192.560), 0.003*
				^b^2.030 (0.191–21.538), 0.557	^**b**^20.073 (2.004–201.063), 0.011*
**Dominant model**					
G/G	60 (80.0%)	33 (44.0%)	66 (88.0%)		
G/A + A/A	15 (20.0%)	42 (56.0%)	9 (12.0%)	^**a**^1.833 (0.747–4.497), 0.186	^**a**^9.333 (4.060–21.456), <0.0001*
				^**b**^1.797 (0.711–4.547), 0.216	^**b**^15.184 (5.387–42.795), <0.0001*
**Recessive model**					
G/A + G/G	72 (96.0%)	63 (84.0%)	74 (98.7%)		
A/A	3 (4.0%)	12 (16.0%)	1 (1.3%)	^**a**^3.083 (0.313–30.336), 0.334	^**a**^14.095 (1.783–111.421), 0.012*
				^**b**^1.906 (0.180–20.155), 0.592	^**b**^11.444 (1.289–101.618), 0.029*
**Allele model**					
G	132 (88.0%)	96 (64.0%)	140 (93.3%)		
A	18 (12.0%)	54 (36.0%)	10 (6.7%)	^**a**^1.909 (0.850–4.286), 0.117	^**a**^7.875 (3.822–16.227), <0.0001*
				^**b**^1.751 (0.759–4.041), 0.189	^**b**^9.294 (4.185–20.638), <0.0001*

OR, odds ratio; AOR, Adjusted odds ratio

^a^**, OR (95% CI);**

^**b**^, AOR **(95% CI);** AA, Alopecia areata

* Statistical significance was considered at *p* value ≤0.017 due to Bonferroni’s correction for multiple testing., values in bold indicates significant values: CI, confidence interval; Hardy- Weinberg equilibrium in control; χ^2^ = 1.531, p = 0.216.

#### 3.3.1. TNF-α-308 (G/A) gene polymorphism (rs1800629) frequency in AA and vitiligo patients and healthy controls

The results showed that there was no significant difference for the G/G genotype between AA and vitiligo cases and HCs **(Table 4)**. The difference in the distribution of G/G genotype and both G/A and A/A genotypes in AA patients was not significant (*p* = 0.264 and 0.557 respectively). Statistical significance was considered at *p*≤0.017 due to Bonferroni's correction for multiple testing.

In AA patients, no risk was associated with G/A (AOR = 1.761, 95%CI = 0.652–4.759, *p* = 0.264), or A/A genotypes (AOR = 2.030 95%CI = 0.191–21.538), *p* = 0.557) compared with the normal G/G allele. Also, no significantly increased risk was reported in the dominant (AOR = 1.797, 95%CI = 0.711–4.547, *p* = 0.216) or recessive genetic model analyses (AOR = 1.906, 95%CI = 0.180–20.155, *p* = 0.592). Similar results were observed for A allele *vs*. G allele analysis (AOR = 1.751, 95%CI = 0.759–4.041, *p* = 0.189) **(Table 4)**.

Vitiligo patients had a higher frequency of the G/A and A/A genotypes (*p*<0.0001 and = 0.011 respectively) compared with HCs. The detailed results are shown in **Table 4.** According to the different genetic models, an association was found between vitiligo and the rs1800629. The genotypic model showed that vitiligo patients who had the mutant G/A and A/A genotypes were at increased risk of vitiligo (AOR = 14.274, 95%CI = 4.661–43.708, *p*<0.0001 and AOR = 20.073, 95%CI = 2.004–201.063, *p* = 0.011 respectively) **(Table 4).**

Similarly, a significantly increased risk was noted with the analysis of the dominant genetic model (AOR = 15.184, 95% CI = 5.387–42.795, *p*<0.0001) and in patients with A allele *vs*. G allele (AOR = 9.294, 95% CI = 4.185–20.638, *p*<0.0001) **(Table 4).** The genotype distributions of rs1800629 in HCs agreed with those expected by the Hardy–Weinberg equilibrium (*p* = 0.216).

#### 3.3.2. The relation between TNF-α -308 (G/A) gene polymorphism and clinical characteristics in AA patients

In AA patients, mutant genotypes (A/A and G/A) were significantly more prevalent in females than males (*p* = 0.002). Also, they were associated with the presence of frontal lesions of the scalp (*p* = 0.001). As regards the SALT score, A/A genotype was associated with a moderate grade of severity (*p*<0.0001) **(Table 5).**

**Table 5:** Relation between TNF-α-308-G/A and clinical characteristics in alopecia areata patients.

		G/G		G/A		A/A		*p*-value
		N	%	N	%	N	%	
**Sex**	Male	30	50.0%	0	0.0%	0	0.0%	0.002*
	Female	30	50.0%	12	100.0%	3	100.0%	
**Family history**	Positive	3	5.0%	0	0.0%	0	0	0.677
	Negative	57	95.0%	12	100.0%	3	100.0	
**Course**	Stable	9	15.0%	3	25.0%	0	0.0%	0.512
	Progressive	51	85.0%	9	75.0%	3	100.0%	
**Clinical**	Single	24	40.0%	6	50.0%	0	0.0%	0.287
	Multiple	36	60.0%	6	50.0%	3	100.0%	
**Site of lesions**								
**Frontal**	No	33	55.0%	0	0.0%	0	0.0%	0.001*
	Yes	27	45.0%	12	100.0%	3	100.0%	
**Lateral**	No	36	60.0%	6	50.0%	3	100.0%	0.287
	Yes	24	40.0%	6	50.0%	0	0.0%	
**Back**	No	36	60.0%	6	50.0%	3	100.0%	0.287
	Yes	24	40.0%	6	50.0%	0	0.0%	
**Body**	No	51	85.0%	9	75.0%	3	100.0%	0.512
	Yes	9	15.0%	3	25.0%	0	0.0%	
**SALT score**								
**S**	0	2	3.3%	0	0.0%	0	0.0%	<0.0001*
	1	34	56.7%	6	50.0%	0	0.0%	
	2	21	35.0%	0	0.0%	3	100.0%	
	3	0	0.0%	2	16.7%	0	0.0%	
	4	3	5.0%	4	33.3%	0	0.0%	
**B**	0	54	90.0%	9	75.0%	3	100.0%	0.278
	1	6	10.0%	3	25.0%	0	0.0%	
**Grade**	Mild	36	60.0%	6	50.0%	0	0.0%	<0.0001*
	Moderate	21	35.0%	1	8.30%	3	100.0%	
	Severe	3	5.0%	5	41.7%	0	0.0%	
		**G/G**		**G/A**		**A/A**		***p*-value**
		**Median**	**Range**	**Median**	**Range**	**Median**	**Range**	
**Age (years)**		11	2.5–55	26.5	2–62	4	4–4	0.055
**Disease duration (Months) Mean±SD**		15	0.25–204	7.5	1–72	6	6–6	0.642

* Statistical significance was considered at *p* value ≤0.05; **SALT**, Severity of Alopecia Tool. The reference genotype was the G/G genotype.

#### 3.3.3. The relation between TNF-α-308(G/A) polymorphism and clinical characteristics in vitiligo patients

There was no statistically significant difference between vitiligo patients with normal genotype and those with mutant genotypes for sex, duration of the disease, clinical types, or VASI score. The A/A genotype was significantly associated with age (*p* = 0.007) and the presence of lesions in the trunk (*p* = 0.009) (**Table 6).**

**Table 6:** Relation between TNF-α-308- G/A and clinical characteristics in vitiligo patients.

		**G/G**		**G/A**		**A/A**		***p*-value**
		**N**	**%**	**N**	**%**	**N**	**%**	
**Sex**	Male	6	18.2%	6	20.0%	3	25.0%	0.880
	Female	27	81.8%	24	80.0%	9	75.0%	
**Clinical type**	Segmented	6	18.2%	9	30.0%	3	25.0%	0.106
	Non-segmented	24	72.7%	12	40.0%	6	50.0%	
	Mixed	3	9.1%	9	30.0%	3	25.0%	
**Site of lesions**								
**Face/Neck**	No	21	63.6%	12	40.0%	6	50.0%	0.170
	Yes	12	36.4%	18	60.00%	6	50.0%	
**Upper Limb**	No	6	18.2%	9	30.00%	6	50.0%	0.104
	Yes	27	81.8%	21	70.0%	6	50.0%	
**Lower Limb**	No	6	18.2%	12	40.0%	3	25.0%	0.151
	Yes	27	81.8%	18	60.0%	9	75.0%	
**Trunk**	No	24	72.7%	21	70.0%	3	25.0%	0.009*
	Yes	9	27.3%	9	30.0%	9	75.0%	
		**G/G**		**G/A**		**A/A**		***p*-value**
		**Median**	**Range**	**Median**	**Range**	**Median**	**Range**	
**Age (Years)**		30	10–40	29.5	6–52	38.5	35–52	0.007*
**Duration (Months)**		36	0.5–276	18	1–420	61.5	1–144	0.927
**VASI score**		0.5	0.05–20	0.98	0.1–7	1.13	0.25–2	0.923

* Statistical significance was considered at *p* value ≤0.05; VASI, Vitiligo Area Scoring Index. The reference genotype was the G/G genotype.

#### 3.3.4. Differences in serum TNF-α level with the different genotypes in AA and vitiligo patients

The results of the current study have revealed that in AA patients, the G/A and A/A genotypes caused a significant increase in sTNF-α levels compared with the G/G genotype (*p*<0.0001 and 0.003 respectively). While in vitiligo patients, those with TNF-α-308 G/G genotypes exhibited significantly lower sTNF-α levels than those with G/A genotypes (*p*<0.0001). In both AA and vitiligo patients, the A allele was associated with a significantly higher sTNF-α level than the G allele (*p*<0.0001) **(Table 7).**

**Table 7:** Differences in TNF-α serum level according to the difference in genotypes among alopecia areata and vitiligo patients.

Disease		TNF-α serum level	
Alopecia areata		Median	Range
**Genotype**	**G/G**	8.3	7.7–9.2
	**G/A**	11	9.3–14.7
	**A/A**	25.3	25.3–25.3
***p*-values**			
G/G vs. G/A	<0.0001*		
G/A vs. A/A	0.858		
G/G vs. A/A	0.003*		
**Allele**	**G**	8.4	7.2–16.8
	**A**	14. 7	9.1–25.3
***p*-value**	<0.0001*		
**Vitiligo**		**Median**	**Range**
**Genotype**	**G/G**	6.2	5.1–6.4
	**G/A**	8.9	8.3–12.3
	**A/A**	8.1	7.9–8.3
***p*-values**			
G/G vs. G/A	<0.0001*		
G/A vs. A/A	0.116		
G/G vs. A/A	0.130		
**Allele**	**G**	6.3	4.1–16.3
	**A**	8.4	6.3–16.3
***p*-value**	<0.0001*		

*Significant

The reference genotype was the G/G genotype.

### 3.4. *In silico* effect of rs1800629 SNP on TNF-α gene

**Fig 2:**
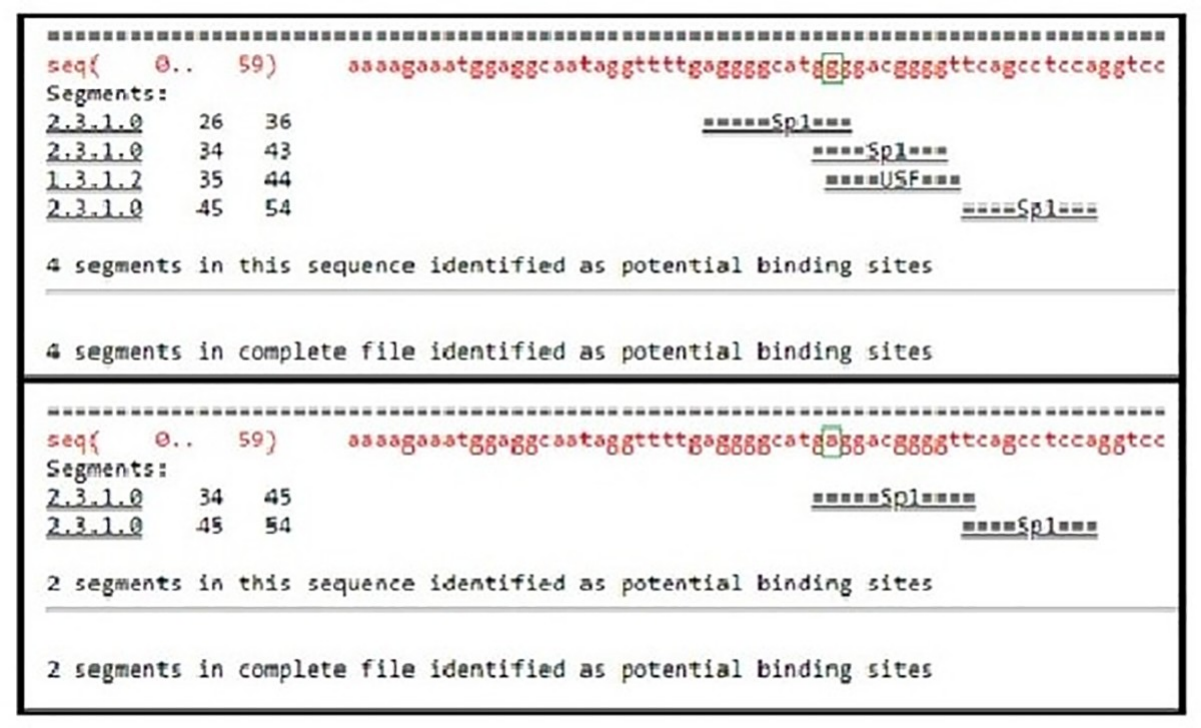
Using Alibaba software, transcription factors binding sites in the normal TNF-α gene sequence were found to be four, three binding sites for specificity protein 1 (SP1) transcription factor and one binding site for upstream transcription factor (USF) transcription factor; while in the mutant sequence, due to the tumor necrosis factor (TNF)-α-308 G/A gene polymorphism (rs1800629), there were two SP1 transcription factor binding sites.

**Fig 3:**
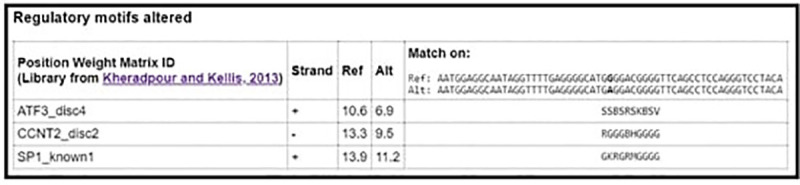
HaploReg v4.1 software predicted an alternation on binding sites of specificity protein 1 (SP1), activating transcription factor 3 (ATF3), and cyclin T2 (CCNT2) transcription factors due to tumor necrosis factor (TNF)-α-308 G/A gene polymorphism (rs1800629).

**Fig 4:**
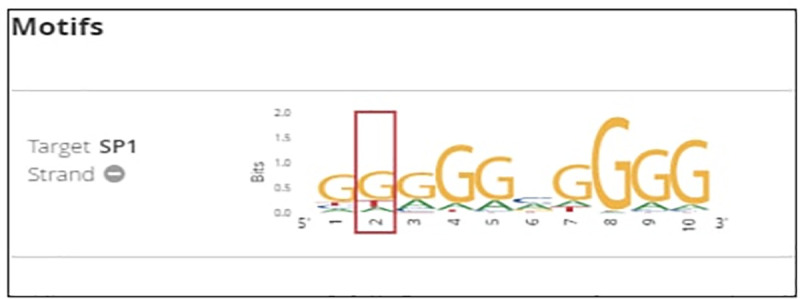
Regulaome DB prediction of affected transcription factor due to the tumor necrosis factor (TNF)-α-308 G/A gene polymorphism (rs1800629), the binding sites of specificity protein 1 (SP1) are altered.

From NCBI database the rs1800629 SNP is found in human at chromosome 6 certainly upstream to TNF*-α* gene in which G nucleotide was replaced by A. According to the *in silico* analysis, this SNP could affect binding sites of some predicted TFs as follows: Alibaba predicted an effect on the binding sites of SP1 (Specificity Protein 1) and USF (Upstream Transcription Factor) (**Fig 2),** HaploReg predicted an effect on the binding sites of SP1, ATF3 (Activating Transcription Factor 3), and CCNT2 (cyclin T2) (**Fig 3)** and Regulome DB predicted an effect on the binding sites of SP1 **(Fig 4).** On the other hand Regulome DB and HaploReg predict that rs1800629 SNP affects many tissues including blood and skin (fibroblast, melanocytes, keratocytes).

## 4. Discussion

Some genetic risk factors are common between AA and vitiligo, suggesting sharing similar pathogenesis [3]. The current case-control study aimed to detect the sTNF-α level and the TNF-α-308(G/A) gene polymorphism in AA and vitiligo patients and to determine their relation with disease duration and severity.

Alterations in TNF-α levels lead to changes in the normal growth of the hair follicle that was found to be associated with the development of AA [12]. As reported in this study, sTNF-α levels in AA patients were significantly raised when compared to HCs. The same results were obtained in earlier studies [22, 23]. Moreover, **El-Tahlawi *et al*.** have stated that biopsies from skin lesions of AA patients had a significantly higher level of TNF-α than that in biopsies from non-lesional skin and controls' biopsies [24].

This study revealed that the difference in sTNF-α levels had no effect on the clinical types of AA (single patch or multiple patches) which is in accordance with **Atwa *et al***. [22]. Also, we found that the sTNF-α levels had no association with disease severity among AA patients. For this point, some previous reports were parallel to ours [23, 25], whereas others were not [22]. Generally, it was expected to reach the conclusion that the disease severity in AA patients is not linked to the sTNF level considering the numerous sophisticated factors that control the disease severity in any person [26].

The current study has revealed that the sTNF-α levels were significantly higher in vitiligo patients than in HCs. Some previous studies [27, 28] supported our findings while others opposed it [29]. A previous study had suggested that melanocyte death in vitiligo patients could be initiated by the increased TNF-α levels [7]. Some clinical trials, that have used anti-TNF-α agents for the treatment of vitiligo, have reported a gradual improvement of illness that signified the involvement of TNF-α in vitiligo pathogenesis [30, 31].

There are numerous polymorphisms associated with AA [32]. In the present study, no association was reported between the presence of the mutant genotypes (G/A and A/A) and AA disease. These results are consistent with **El Sayed *et al*.,** from Egypt [33]. Also, TNF-α-308A allele was more frequent in AA patients than in HCs; however, this difference was statistically non-significant. Similar findings were reported by **Galbraith and Pandey** [34].

Polymorphisms of variable immune system genes have been found to play a role in the development of vitiligo. In the current study, the rs1800629 was more frequent among the vitiligo patients than in the HCs. Similar findings were reported by, **Al-Harthi *et al*.,** from KSA [35]. In vitiligo patients, our study suggested that the mutant genotypes, G/A and A/A, are associated with higher risk for vitiligo, whereas G/G genotype might confer resistance against the disease. An Iranian study was in line with our study [36]. However, this does not agree with previous reports demonstrating that TNF-α-308 SNP did not confer risk or vitiligo susceptibility [11, 37]. These contradictory reports maybe because of the differences in ethnicity of the studied populations.

We also found that the TNF*-*α-308A allele was significantly associated with vitiligo compared with HCs. This concurs with the results obtained from KSA, and Iran [35, 36] and with a meta-analytic study which reported that the TNF-α-308A allele was significantly associated with vitiligo in Middle Eastern populations [13].

This study has demonstrated the considerable role of TNF-α promoter polymorphisms on the sTNF-α as we reported a significant rise in the sTNF-α level with the mutant genotypes (G/A and A/A) in comparison to the normal G/G genotype which might be playing a central role in vitiligo pathogenesis. These results agree with earlier reports [7, 28]. Also, the TNF-α-308A allele was much more associated with higher sTNF-α than the common G allele. Similar findings were outlined by **Kroeger and colleagues** [38].

The results suggest that serum TNF-α levels are significantly associated with the clinical characteristics of AA patients, though not with those of vitiligo patients. However, the rs1800629 was significantly associated with susceptibility to vitiligo, but not with AA. This discrepancy could be justified by the fact that the development of autoimmune diseases, the differences in disease severity, the distribution of lesions and other clinical characteristics are affected by multiple factors which include genetic and environmental factors.

Several studies have now demonstrated that this polymorphism affects transcription factor binding and enhances transcription from the TNF2 promoter in monocytic and lymphocytic cell lines [39]. Another objective of this study was to use bioinformatics to analyze the SNP (rs1800629) and to determine its effect on the levels, regulation and interactions of their relevant proteins. The SP1 transcription factor binding site disruption by the rs1800629 SNP was predicted by all of the used softwares; Regulome DB, HaploReg and Alibaba softwares. It was reported that an intact SP1 binding site is required for virus-induced expression of TNF-α gene with enrollment of TNF-α enhancer complex that contains NFAT, ATF-2/Jun, and SP1. Therefore, the binding between the TNF-α promoter and SP1 in the environment of the enhancer complex, either by overexpression of SP1 together with the other transactivators or by virus-specific induction of SP1, stimulates the transcription of the TNF-α gene [40].

According to this relation between SP1 and TNF-α, with the presence of this SNP that affects the binding site of SP1, a decrease in the TNF-α gene expression could be the expected net result. However, in this study, an association between rs1800629 SNP and high sTNF-α was reported. This can be clarified by the reports confirming that gene activation by SP1 can be affected by positive and negative relations with other transcription factors [41]. Also, SP1-dependant transcription depends on the collaboration of other transcriptional cofactors and activators present in the complex promoters [42].

Regulome DB and HaploReg predict that rs1800629 SNP affects many tissues including blood and skin (fibroblasts, melanocytes, keratocytes), which is consistent with the pathological changes of vitiligo and AA that involve melanocytes and keratocytes.

The haploReg software was the only tool to predict distortion of ATF3 binding site with presence of the rs1800629 SNP. ATF3 is a member of the activating transcription factor (ATF) family of proteins [43]. Previous studies have shown that regular transcription of the TNF-α was controlled by ATF3 by inhibiting the AP-1 binding to its promoter to obstruct the transcription activation by NF-κB [44, 45]. Furthermore, primary macrophages from *Atf3*^−/−^ mice show increased expression of TNF-α, IL-6, and IL-12, demonstrating that ATF3 is a negative controller of immune responses [46] and explains the observed increase in TNF-α serum level with the mutant genotypes reported in the current study.

## 5. Conclusion

From this study we have concluded that the rs1800629 SNP was a risk factor for vitiligo, but not AA, among Egyptian subjects. The *in silico* analysis has verified the molecular effect of the rs1800629 SNP on TNF-α transcription with a potential subsequent effect on the pathogenesis of many autoimmune diseases. Further studies evaluating cytokines in tissues and peripheral blood on extended samples will help to clarify the pathogenesis of these diseases.

## Abbreviations

AA: Alopecia areata
AOR: Adjusted Odds ratio
ATF3: Activating Transcription Factor 3
CCNT2: cyclin T2
CI: confidence intervals
HCs: healthy controls
HF: hair follicle
HWE: Hardy-Weinberg equilibrium
IQR: interquartile range
KS: Kolmogorov-Smirnov test
LD: linkage disequilibrium
MHC: major histocompatibility complex
NCBI: National Center of Biotechnology Information
OR: Crude Odds ratio
PUVA: psoralen and ultraviolet A
RT-PCR: real time-PCR
SALT: Severity of Alopecia Tool
SD: standard deviation
SNPs: single-nucleotide polymorphisms
SP1: Specificity Protein 1
sTNF-α: serum TNF-α
TFs: transcription factors
TNF-α: Tumor necrosis factor-α
USF: Upstream Transcription Factor
VASI: Vitiligo Area Scoring Index
